# Unplanned Pregnancy and Depressive Symptoms during the COVID-19 Pandemic

**DOI:** 10.3390/ijerph20010652

**Published:** 2022-12-30

**Authors:** Gilberto Assunção Costa Júnior, Adriana Sousa Rêgo, Andressa Pestana Brito, Poliana da Silva Rêgo Furtado, Thayla Thais Jatahy Pereira, Lucas Frota Beckman, Yuri Alfredo Araujo Mendonça, Cristina Nitz da Cruz, Magali Kelli Nitz, Márcia Rodrigues Veras Batista, Márcio Anderson Sousa Nunes, Janaina Maiana Abreu Barbosa, José Márcio Soares Leite, Ângela Falcai, Marcos Antônio Barbosa Pacheco, Cristina Maria Douat Loyola, Maria Raimunda Chagas Silva, Wellyson da Cunha Araújo Firmo, Flor de Maria Araujo Mendonça Silva

**Affiliations:** 1 Postgraduate Program in Management of Health Programs and Services, Ceuma University, Campus Renascença, São Luís 65075-120, Maranhão, Brazil; 2 Health Sciences Center, State University of the Tocantina Region of Maranhão, Campus Imperatriz, Imperatriz 65900-000, Maranhão, Brazil

**Keywords:** COVID-19, depression, mental health, pregnant women, public health

## Abstract

This is a cross-sectional study conducted with pregnant women who underwent prenatal care at basic health units in São Luís City, Maranhão State, Brazil. The authors used a semistructured questionnaire to assess the socioeconomic, demographic, and clinical characteristics of pregnant women as well as the Edinburgh Scale to investigate depressive symptoms. In order to assess the association between the explanatory variable and the outcome variable, Poisson logistic regression was performed with statistical significance at *p* < 0.05. A total of 205 women were interviewed, most aged between 18 and 29 years (66.83%). Of this total, 74.63% had not planned their pregnancy and 26.67% had depressive symptoms. The variables unplanned pregnancy (PR = 1.41; CI = 0.99–2.00; *p* = 0.05) and not undergoing psychological counseling (PR = 1.42; CI = 0.51–0.83; *p* ≤ 0.01) correlated with depressive symptoms during pregnancy. It is thus possible to link the variables unplanned pregnancy (*p* > 0.05) and not undergoing psychological counseling (*p* = 0.001) to depression. Therefore, it is important to monitor the mental health of pregnant women, especially in situations of vulnerability.

## 1. Introduction

According to the concept of a health condition developed by the World Health Organization (WHO), the puerperal pregnancy cycle is a circumstance in the life of pregnant women that follows a period of fertility and that can be desired and planned or surprising and unforeseen, with feelings of ambivalence and non-acceptance. In the same way, another circumstance in a woman’s life follows: motherhood, which evokes a double responsibility in the mother–child binomial [1].

Insecurity in dealing with the feelings, emotions, and care required during pregnancy and during the postpartum period leads to inadequacy in the performance of the maternal role. Studies indicate that the performance, adaptation, and development of maternal care are closely linked by individual, cultural, and family aspects, which, when not identified, affect a pregnant woman’s feeling of acceptance towards motherhood [2,3].

With this, research is still observed that shows that mental health problems are among the most commonly reported pregnancy complications, affecting up to 20% of pregnant women during and after pregnancy. In the United Kingdom, some mental health problems (anxiety crises, decreased mood, and panic) are the main causes of maternal death, with cases recorded up to the first postpartum year [4].

The Brazilian Ministry of Health declares that family planning must be treated within the context of reproductive rights, with the main objective of guaranteeing women the right to have or to not to have children [5]. In fact, this phenomenon has other contours in developing countries. Even with the right to plan pregnancy, the birth rate has increased due to several factors, including exponential increases in offspring, nonadherence to contraceptive methods, and a lack of sexual and reproductive education in underdeveloped countries [6].

Data from the Brazilian National Demographic and Health Survey (PNDS) carried out in 2006 indicate that of the total number of births in recent years, only 54% were planned. Of the 46% unplanned births, 28% were expected to occur at a different time, and 18% were definitely not wanted. Unintended pregnancy has become a problem that requires careful attention to prevention [7].

Although unplanned pregnancy is an indication of a possible public health problem, given its frequency and percentages, few recent epidemiological and/or prevalence studies were found on the relationship between unplanned pregnancy and depressive symptoms. Therefore, there is a need for studies that analyze the health of pregnant women throughout the entire gestational period and their main living arrangements, routines, and cultures [8].

The gestational period comprises a high rate of injuries associated with mental disorders. Moreover, we have been experiencing a pandemic scenario over the last two years. In this context, the present article investigates the association between unplanned pregnancy and depressive symptoms in pregnant women assisted in primary care in São Luís City, Maranhão State, Brazil, during the COVID-19 pandemic.

## 2. Materials and Methods

The methodological design consisted of a cross-sectional analytical approach nested within the Pregnant Women Cohort under Opinion No. 3258471. The study used a nonprobabilistic sample of pregnant women over 18 years of age who lived in São Luís City, Maranhão State, and who underwent prenatal care at basic health units (UBS).

São Luís City has a population of 1,014,837 and an area of 583.063 km^2^. Of this area, 157,656 km^2^ are in the urban perimeter of Maranhão State. The Human Development Index (HDI) of the city is 0.768 [9].

Sample size calculation considered that the prevalence of depressive pregnant women in Maranhão State was 20.5% [8]. Thus, 205 pregnant women would be needed to achieve statistical representation. Pregnant women aged ≥ 18 years with a single fetus and who received prenatal care at basic health units were included. The study did not include pregnant women who declared themselves unable to answer the questions in the questionnaire.

The data collection procedure included a semistructured questionnaire with closed questions about the socioeconomic, demographic, and clinical data of the pregnant women who had received prenatal care in primary care. The explanatory variables used were age (18–29; 30–41), race/color (black, white, brown/other), marital status (living with a spouse or partner and not living with a spouse or partner), number of household members (1 to 4 or 5 to 8), education (elementary school and high school), government benefits (no or yes), occupation (no or yes), childbirth (primiparous or multiparous), abortions (no or yes), planned pregnancy (no or yes), accepted pregnancy (no or yes), physical activity before and during pregnancy (no or yes), smoking (no or yes), alcohol consumption (no or yes), psychological counseling (no or yes), and symptoms of COVID-19 (with symptoms and without diagnosis/without symptoms and without diagnosis and with symptoms and diagnosis/without symptoms and with diagnosis).

The instrument used to investigate depression symptoms was the Edinburgh Postpartum Depression Scale (EPDS), which was validated in Brazil by Santos, Martins, and Pasqual [10]. It is a self-report questionnaire composed of ten items that assess the presence and intensity of depressive symptoms in the previous seven days using a Likert scale that ranges from 0 to 3. The version validated in Brazil considers a score ≥ 10 for depressive symptoms as the most appropriate cutoff point for the country context [10]. In a recent validation study with 140 pregnant women, it was used from the first trimester to 6 (six) weeks after delivery [11].

**Figure 1:**
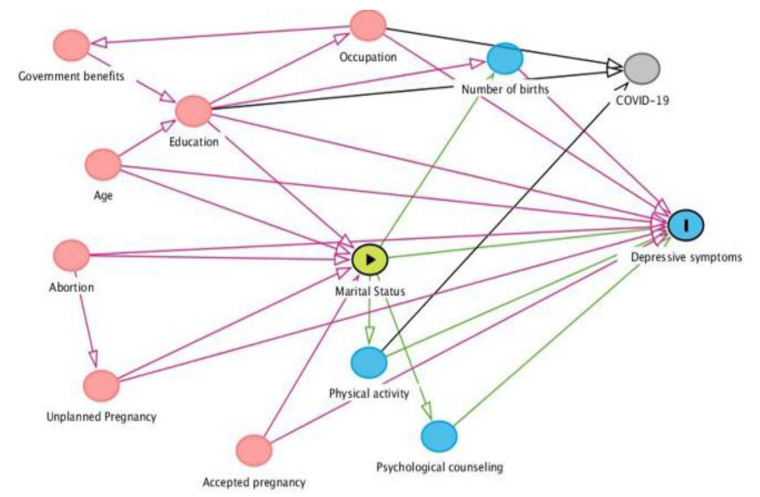
Theoretical model to assess the association of depression symptoms.

A theoretical model was built to evaluate the covariates that would be used as controls in a study of the association between the explanatory variable and the outcome (depressive symptoms during pregnancy). For that, directed acyclic graphs (DAG) were developed in the Dagitty 2.2 program (www.dagitty.net4, accessed on 13 September 2021) (Figure 1).

The non-probabilistic sample and the calculation of the sample size were performed using the statistical program G * Power 3.1 by considering a significance level (α) of 5%, a test power of 80%, and a tolerable error of 4%, plus 10% for possible losses. The calculation determined that 205 pregnant women would be a necessary, adequate number of pregnant women in each of the categories of the study variables to achieve statistical significance.

Statistical analysis was performed using STATA 15.0 software (StataCorp., College Station, TX, USA). To assess the association between the explanatory variable (pregnant women who received prenatal care during the COVID-19 pandemic) and the response variable (pregnant women who presented with depressive symptoms), the Chi-square test was used for independent samples. Inferential analytical statistics were first performed using Poisson logistic regression to assess the Prevalence Ratio (PR) with robust variance adjustment for events that presented *p* < 0.20 in the univariate analysis; events with *p* < 0.10 were included in the final multivariate model with the respective Prevalence Ratios (PR), 95% Confidence Intervals (95% CI), and statistical significance of *p* < 0.05.

## 3. Results

The sample for the present study consisted of 205 pregnant women. With regard to socioeconomics and demographics, the mean age was 26.2 years (SD ± 6.11), and the average number of members in each household was 3.41 (SD ± 1.32). Most women were aged between 18 and 29 years (66.83%); 58.54% declared themselves to be brown; 74.15 lived with a partner; 89.76% had elementary school education; 63.41% received government benefits; and 67.32% had no occupation. As for reproductive characteristics, 95.12% were multiparous, 74.63% had not planned their pregnancy, and 10.75% did not accept their pregnancy. Regarding lifestyle, 67.80% reported not engaging in physical exercise before pregnancy; during pregnancy, this percentage increased to 89.27%. A total of 0.49% of the pregnant women reported smoking and drinking alcohol. Regarding psychological counseling, 92.20% did not receive it, and 26.67% had depressive symptoms. With regard to COVID-19, 9.5% had a positive diagnosis (Table 1).

The age variable had percentages of 66.83% (18–29 years) and 33.17% (30–41 years), with the percentages for the presence of depressive symptoms in the following categories being 51.86% (18–19 years) and 52.95% (30–41 years), with no statistical significance (*p* = 0.95).

After analyzing the theoretical model, the variables of unplanned pregnancy (PR = 1.41; CI = 0.99–2.00; *p* = 0.05) and not undergoing psychological counseling (PR = 1.42; CI = 0.51–0.83; *p* ≤ 0.01) correlated with depressive symptoms during pregnancy (Table 2).

## 4. Discussion

In the present study, unplanned pregnancy and not undergoing psychological counseling was correlated with depressive symptoms during pregnancy. In a study carried out in Rio Grande do Sul, Brazil, in which 2687 puerperal women were interviewed, 14% were identified as having depression, and it was observed that planning the pregnancy was a protective factor that reduced the risk by 27% [12]. In a cohort study conducted in Acre, Brazil, with 461 pregnant women, no association was found between unplanned pregnancy and common mental disorders [13]. Although there are differences between these results, it is important to value family planning as a risk factor for mental health, and some studies show a high prevalence of non-high pregnancy planning [14,15].

Unplanned pregnancy is any pregnancy that is not planned by the couple or the woman. It can occur due to omission, carelessness, or lack of knowledge of contraceptive methods, which can cause the couple to experience mental suffering, instability in the relationship, and insecurity [6,16,17].

Pregnancy is a phase that interferes with family routine. Not planning it can thus lead to the development of initial symptoms of mental disorders related to mood, anxiety, and stress, with possible complications in the postpartum period [18].

The present study points out a high percentage of unplanned pregnancies, in which 31.2% of the women had depressive symptoms. A cohort study carried out with 1,121 pregnant women aged 18 to 49 years enrolled in the Family Health Strategy (ESF) of Recife-PE found a 60.2% frequency of unplanned pregnancies, in which 25.9% of the women presented depressive symptoms after childbirth [19]. These data show the importance of investigating depressive symptoms during pregnancy and offering mental health services to pregnant women during prenatal care.

In the present study, psychological counseling was a protective factor against depressive symptoms during pregnancy. These data indicate that tracking depressive symptoms during prenatal care can provide visibility of this phenomenon, enabling the possibility of intervention.

A multinational cross-sectional study carried out with 3545 pregnant women in Ireland, Norway, Switzerland, the Netherlands, and the United Kingdom during the COVID-19 pandemic detected depressive symptoms in 15% (n = 533) of them [20]. These symptoms may have been caused by restrictive measures and changes in mental health care, increasing the suffering of these pregnant women [21,22].

Another survey carried out with 1,832 pregnant women showed that 446 (24.3%) women had a score above the Edinburgh scale cutoff point at least once during the trimester. Of these, only 1.7% sought mental health professionals, demonstrating low adherence to mental health services [23].

In a study with 76 pregnant women, 47 participated in psychological prenatal care, and 23.68% were at risk for postpartum depression (PPD). It is noteworthy that only 10.64% of the women in the intervention group were at risk for PPD. In contrast, 44.83% of the women in the sample did not receive psychological prenatal care. These women showed a higher prevalence of depressive symptoms since psychological counseling is a protective factor [24].

Given the above, it is essential to revitalize psychological counseling programs during pregnancy and postpartum for the consolidation and improvement of health policies [25,26]. However, basic health units in Brazil follow the guidelines of the Ministry of Health, privileging actions aimed at the biological dimension. This perpetuates a medical model of care that does not sufficiently address psychosocial aspects.

The present study showed that although the variable COVID-19 infection did not correlate with the outcome of depressive symptoms in pregnant women, the prevalence of depressive symptoms was notably high. Research such as that of Ceulemans et al. [20] also shows a high prevalence of this condition. Therefore, it is important to monitor the mental health of pregnant women, especially in situations of vulnerability.

### Limitations and Strong Points

Research limitations were caused due to sanitary conditions on that date. The care network was under health restrictions (COVID-19), making data collection impossible for a while. To solve the sampling error, a sample calculation was performed based on the prevalence of gestational depression by considering a test power of 80%. To ensure a more robust sample, pregnant women were offered the possibility of participating in a virtual host group that benefited them with knowledge and information about COVID-19, appointments, and virtual assistance from professionals: nurses, physiotherapists, doctors, psychologists, and nutritionists.

The population of pregnant women was considerably challenging, as part of the sample consisted of pregnant women who had not completed high school, directly affecting the interview format and approach. The support and reference team was mostly made up of doctors and nurses, not allowing the focus to be on mental health and/or to be outside of a purely biomedical model.

A strong point of the research was the early identification of the presence of signs of depression in pregnant women and the food and nutritional insecurity experienced by them. In these cases, the pregnant woman was referred to a psychologist and referred to a state government assistance program for pregnant women for benefits in addition to being enrolled in food reuse workshops. Investigating food and nutrition insecurity was a strength of the study, as lack of food is an important determinant to be studied in conjunction with mental health.

It is necessary that the mental health of pregnant women be monitored during prenatal care, not only due to the hormonal changes caused by the gestational cycle, but also due to the context of social inequality that can worsen and reflect emotional aggravation that can be extended into the postpartum period and in the child.

## 5. Conclusions

The results of this study have the potential to encourage and develop management strategies, enabling an increase in educational activities for the community of pregnant women and the inclusion of the psychological prenatal services in basic health units and in all health care coverage.

Failure to carry out family planning is associated with depression and is a variable that should receive attention in the assistance and monitoring of fertile women, as most pregnant women reported not carrying out family planning. However, pregnant women who have not undergone family planning should receive special attention, as it is a marker that can impact mental health since not receiving psychological follow-up was associated with depression. The main criticism is also related to the poor visibility of the mental health professionals in the coverage of care received during pregnancy and its main nuances.

## Figures and Tables

**Table 1:** Distribution of sociodemographic, demographic, lifestyle, and reproductive variables and depressive symptoms of pregnant women followed up with during prenatal care at basic health units in São Luís City, Maranhão State, Brazil, in 2022.

Variable	n° (205) %	Edinburgh Postpartum Depression Scale (EPDS)		*p*-Value
		Absencia of Symptons n° (%)	Presence of Symptons n° (%)	
**Age**				0.95
18–29	137 (66.83)	65 (48.14)	72 (51.86)	
30–41	68 (33.17)	32 (47.05)	36 (52.95)	
**Marital status**				0.33
Living with a spouse or partner	152 (74.15)	64 (42.10)	88 (57.90)	
Not living with a spouse or partner	53 (25.85)	33 (62.26)	20 (37.74)	
**Education**				<0.001
Elementary school	184 (89.76)	82 (44.56)	102 (55.44)	
High school	21 (10.24)	15 (71.42)	6 (28.58)	
**Government benefits**				0.05
No	75 (36.59)	29 (38.66)	46 (61.34)	
Yes	130 (63.41)	68 (52.30)	62 (47.70)	
**Occupation**				0.11
No	138 (67.32)	60 (43.47)	78 (56.53)	
Yes	67 (32.68)	37 (55.22)	30 (44.78)	
**Childbirth**				0.26
Primiparous	10 (4.88)	4 (40.00)	6 (60.00)	
Multiparous	195 (95.12)	94 (48.20)	101 (51.80)	
**Abortions**				0.19
No	161 (78.54)	77 (47.82)	84 (52.18)	
Yes	44 (21.46)	20 (45.45)	24 (54.55)	
**Planned pregnancy**				<0.001
No	153 (74.63)	65 (42.48)	88 (57.52)	
Yes	52 (25.37)	32 (61.53)	20 (38.47)	
**Accepted pregnancy**				0.77
No	22 (10.73)	9 (40.90)	13 (59.10)	
Yes	183 (89.27)	88 (48.08)	95 (51.92)	
**Current practice of physical activity**				0.19
No	183 (89.27)	87 (47.54)	96 (52.46)	
Yes	22 (10.73)	10 (45.45)	12 (54.55)	
**Psychological counseling**				<0.001
No	189 (92.20)	95 (51.91)	94 (48.09)	
Yes	16 (7.80)	2 (12.50)	14 (87.50)	
**COVID-19**				0.18
With symptoms and without diagnosis/without symptoms and without diagnosis	129 (62.93)	60 (46.51)	69 (53.49)	
With symptoms and diagnosis/without symptoms and with diagnosis	76 (37.07)	37 (48.68)	39 (51.32)	

**Table 2:** Unadjusted and adjusted analysis of socioeconomic and demographic variables and factors associated with depressive symptoms in pregnant women followed up with during prenatal care at basic health units in São Luís City, Maranhão State, Brazil, in 2021.

Variable	Unadjusted Analysis			Adjusted Analysis *p* ≤ 0.01		
	PR	CI (95%)	*p*-Value	PR	CI (95%)	*p*-Value
**Marital status**			0.16			
Living with a spouse or partner	1	1		1	1	1
Not living with a spouse or partner	0.65	0.44–0.94	0.02	0.84	0.56–1.24	0.25
**Current practice of physical activity**			0.925			
No	1		1			
Yes	1.01	0.70–1.47				
**Education**			0.35			
Elementary School	1	1	1			
High School	0.51	0.35–1.02	0.35			
**Government benefits**			0.45			
No	1	1	1			
Yes	0.98	0.99–1.65	0.46			
**Planned pregnancy**			0.01			
No	1	1		1	1	
Yes	1.49	1.03–2.16	0.05	1.41	0.99–2.00	0.05
**Psychological counseling**			≤0.01			
No	0.56	0.44–0.71	≤0.01	0.65	0.51–0.83	≤0.01
Yes	1	1		1	1	
**Abortions**			0.90			
No	1	1				
Yes	0.98	0.71–1.34				
**COVID-19**			0.76			
No	1	1				
Yes	1.04	0.79–1.36				
**Planned pregnancy**			0.31			
No	1	1				
Yes	0.94	0.83–1.77				

## Data Availability

The datasets generated or analyzed during this study are available and can be obtained, upon request, from Flor de Maria Araujo Mendonça Silva (e-mail: floragyhn@gmail.com).
